# Mitochondrial DNA Instability and Neuroinflammation: Connecting the Dots Between Base Excision Repair and Neurodegenerative Disease

**DOI:** 10.3390/genes17010082

**Published:** 2026-01-13

**Authors:** Magan N. Pittman, Mary Beth Nelsen, Marlo K. Thompson, Aishwarya Prakash

**Affiliations:** 1 University of South Alabama Health Mitchell Cancer Institute, Mobile, AL 36604, USAmary.beth.nelsen@southalabama.edu (M.B.N.);; 2 Department of Biochemistry and Molecular Biology, University of South Alabama, Mobile, AL 36688, USA

**Keywords:** mitochondrial DNA, damage associated molecular patterns, neuroinflammation, neurodegeneration, base excision repair

## Abstract

Neurons have exceptionally high energy demands, sustained by thousands to millions of mitochondria per cell. Each mitochondrion depends on the integrity of its mitochondrial DNA (mtDNA), which encodes essential electron transport chain (ETC) subunits required for oxidative phosphorylation (OXPHOS). The continuous, high-level ATP production by OXPHOS generates reactive oxygen species (ROS) that pose a significant threat to the nearby mtDNA. To counter these insults, neurons rely on base excision repair (BER), the principal mechanism for removing oxidative and other small, non-bulky base lesions in nuclear and mtDNA. BER involves a coordinated enzymatic pathway that excises damaged bases and restores DNA integrity, helping maintain mitochondrial genome stability, which is vital for neuronal bioenergetics and survival. When mitochondrial BER is impaired, mtDNA becomes unstable, leading to ETC dysfunction and a self-perpetuating cycle of bioenergetic failure, elevated ROS levels, and continued mtDNA damage. Damaged mtDNA fragments can escape into the cytosol or extracellular space, where they act as damage-associated molecular patterns (DAMPs) that activate innate immune pathways and inflammasome complexes. Chronic activation of these pathways drives sustained neuroinflammation, exacerbating mitochondrial dysfunction and neuronal loss, and functionally links genome instability to innate immune signaling in neurodegenerative diseases. This review summarizes recent advancements in understanding how BER preserves mitochondrial genome stability, affects neuronal health when dysfunctional, and contributes to damage-driven neuroinflammation and neurodegenerative disease progression. We also explore emerging therapeutic strategies to enhance mtDNA repair, optimize its mitochondrial environment, and modulate neuroimmune pathways to counteract neurodegeneration.

## 1. Introduction

Cells of the central nervous system are among the most energy-intensive in the human body, relying on a vast network of mitochondria to sustain the high ATP output required for synaptic transmission and cellular maintenance [1,2]. The essential role of mitochondrial DNA (mtDNA) in encoding crucial subunits of the electron transport chain (ETC) directly connects genome integrity to neuronal energy homeostasis [3]. Unlike nuclear DNA, mtDNA lacks protective histones and is located near sites of oxidative phosphorylation (OXPHOS), which continually exposes it to reactive oxygen species (ROS) produced during respiration [3,4,5,6]. Although many oxidative lesions are efficiently repaired or tolerated with minimal impact, persistent or unrepaired mutagenic damage can compromise ETC function and reduce ATP production when a detrimental threshold is reached, and this decline in bioenergetics activates cellular stress responses that can create a self-perpetuating cycle of mitochondrial dysfunction, elevated ROS generation, and progressive mtDNA instability [7,8,9,10,11,12,13]. The long life and post-mitotic nature of neurons prevent the dilution or replacement of damaged mitochondria through cell division. As a result, mtDNA lesions accumulate over time and progressively compromise oxidative phosphorylation, promoting neurodegeneration in disorders such as Alzheimer’s disease and Parkinson’s disease [14,15,16,17,18].

While mitochondria possess additional quality control mechanisms, such as rapid mtDNA turnover, dynamic fission and fusion remodeling, and targeted mitophagy, there is ongoing debate over the necessity of mtDNA repair [4,19,20]. Some groups propose that these quality control systems can compensate for mtDNA damage by eliminating or diluting dysfunctional genomes, reducing the need for robust DNA repair pathways, such as base excision repair (BER) [5,21]. This viewpoint suggests that rapid mitochondrial turnover may limit the long-term consequences of mtDNA lesions.

However, others argue that exclusive dependence on degradation and turnover is energetically expensive and ultimately unsustainable, particularly for long-lived, post-mitotic neurons. Constant mitophagy and biogenesis would present a significant metabolic burden on cells and risk depleting healthy mitochondrial pools [22]. Therefore, a finely tuned balance is required, in which active repair provides a first-line defense against oxidative damage. Among the known cellular DNA repair systems, BER is the only fully characterized and universally accepted functional DNA repair pathway within mitochondria, directly correcting oxidative and other non-bulky lesions, thereby preserving mtDNA integrity and supporting cellular longevity [23,24,25,26,27,28,29,30,31,32,33]. Damage that escapes repair can give rise to not only genomic instability but also to innate immune activation. Notably, unstable or fragmented mtDNA can be released into the cytosol or extracellular milieu, acting as damage-associated molecular patterns (DAMPs) that activate innate immune signaling pathways, such as the cyclic GMP-AMP synthase-stimulator of interferon genes (cGAS-STING) pathway and inflammasomes [34]. Chronic neuroinflammation driven by these signals exacerbates neuronal dysfunction and accelerates neurodegenerative processes.

Our goal in this review is to provide a comprehensive overview of recent advances in understanding the role of BER in maintaining mitochondrial genome integrity in neurons. We examine current perspectives on how BER dysfunction contributes to genetic instability, bioenergetic deficits, and neuroinflammation, and we highlight emerging therapeutic strategies targeting DNA repair and neuroimmune pathways. Finally, we identify outstanding questions and delineate promising directions for future research to interrupt or slow the progression of neurodegeneration.

## 2. Mitochondrial DNA: Structure, Function, and Instability in Neurons

**Figure 1:**
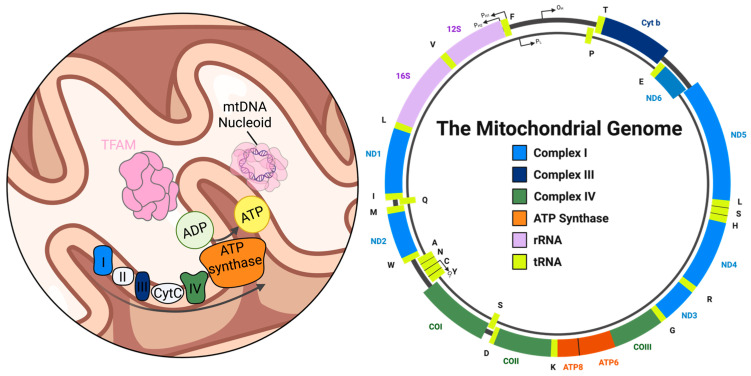
Mitochondrial nucleoid arrangement and genome content. **Left**: Schematic representation of a mitochondrion, highlighting nucleoids tethered to the inner mitochondrial membrane (IMM). A transparent nucleoid visually reveals the mitochondrial DNA (mtDNA). Adjacent to the nucleoid, electron transport chain (ETC) subunits are shown embedded in the IMM. **Right**: The human mitochondrial genome map, showing the distribution of protein-coding genes for ETC complexes I, III, IV, and ATP synthase, plus mitochondrial tRNAs and rRNAs needed for local translation within the organelle.

As a relic of their bacterial ancestry, mitochondria retain a separate genome from the nuclear genome [35,36,37]. Human mtDNA is a double-stranded, circular molecule of approximately 16.5 kb that encodes 13 core subunits of respiratory complexes I, III, IV, and V (ATP synthase), along with two rRNAs and 22 tRNAs, supporting a dedicated mitochondrial translation system that uses a genetic code distinct from the nuclear genome [38,39,40,41] (Figure 1). Despite its essential role, mtDNA contributes only a small fraction of the mitochondrial proteome. More than ~1500 additional proteins, including OXPHOS components and genome maintenance factors, are encoded by nuclear genes, translated in the cytosol, and imported into mitochondria via several distinct pathways. These include the presequence pathway for proteins bearing N-terminal mitochondrial targeting sequences, the carrier pathway for multi-pass inner membrane proteins, and specialized routes for β-barrel and intermembrane space proteins [42,43]. Several dual-localized BER enzymes (including APE1 and specific glycosylases) show dynamic expression and compartmentalization, with oxidative stress enhancing their mitochondrial accumulation to match increased oxidative DNA damage [44,45,46].

Our typical view of DNA organization is shaped mainly by what is observed in the nucleus, where DNA is tightly wrapped around histone octamers to form nucleosomes. These repeating units occur hundreds of thousands of times along each chromosome, creating dense protein shielding and highly ordered chromatin architecture [47,48]. However, mtDNA is packaged in a fundamentally different manner. Instead of nucleosomes, mtDNA is organized into protein–DNA complexes, called nucleoids, which lack histones [3,49,50,51,52]. Mammalian cells contain hundreds to approximately 100,000 copies of mtDNA, with neurons typically enriched in mitochondria [2,53,54]. Even so, total mtDNA copy number per neuron typically ranges from only a few thousand, which is lower than the estimated number of mitochondria per neuron [55]. This likely reflects the heterogeneous distribution of nucleoids, as individual nucleoids typically contain only one or a few mtDNA molecules, and, as a recent preprint reported [56], many axonal or distal neuronal mitochondria appear to lack detectable mtDNA, resulting in regions of the mitochondrial network that are effectively genome-poor [20,56,57,58,59]. This apparent mismatch highlights the decentralized, multi-copy organization of mtDNA, which contrasts sharply with chromosome-wide compaction in the nucleus and provides a more dynamic framework for genome distribution across the mitochondrial network [59].

Each mitochondrial nucleoid forms a compact, highly regulated, and dynamic complex that contains approximately 37 distinct proteins [60]. The central architectural component is mitochondrial transcription factor A (TFAM), which coats and bends mtDNA to promote tight packaging while simultaneously regulating transcription initiation [61,62,63]. Other key mitochondrial replisome proteins are enriched within nucleoids, including Twinkle, the main mtDNA helicase; DNA polymerase gamma (POLG), which synthesizes new mtDNA strands, and the mitochondrial single-stranded DNA-binding protein (mtSSB), which stabilizes single-stranded DNA and promotes processive replication [49]. Collectively, these proteins form the nucleoid’s functional hub, mediating essential genome maintenance and expression.

Nucleoids are typically tethered to the inner mitochondrial membrane, positioning replication and transcription machinery adjacent to the ETC [64] (Figure 1). This localization, while enhancing the translation and membrane integration of OXPHOS proteins, also increases the exposure of mtDNA to an oxidative environment. The inner membrane is the leading site of OXPHOS and the primary source of ROS, making mtDNA much more prone to oxidative damage than nuclear DNA [6,65,66]. Throughout this review, we use ROS to refer primarily to mitochondrial superoxide- and hydrogen peroxide-derived oxidants, while acknowledging that reactive nitrogen species (RNS), such as nitric oxide and peroxynitrite, contribute to similar types of lesions in neuroinflammatory settings [67]. The absence of histones and an open DNA configuration further increases exposure to ROS, lipid peroxidation products, and environmental toxins [6]. Compared to the nucleus, the protective DNA repair activities in mitochondria are limited: while BER is well established, the overall repertoire of glycosylases and other downstream repair enzymes is restricted, and entire pathways such as nucleotide excision repair and mismatch repair are absent or incomplete [25,68,69,70]. However, enzymes traditionally associated with some of these pathways accumulate in the mitochondria and may assist in BER [24].

The consequences of compromised mtDNA maintenance manifest clinically. Pathogenic mutations in mtDNA underlie a range of mitochondrial diseases, such as Leber hereditary optic neuropathy (LHON), mitochondrial encephalomyopathy, lactic acidosis, and stroke-like episodes (MELAS), myoclonic epilepsy associated with ragged red fibers (MERRF), and neurogenic muscle weakness, ataxia, and retinitis pigmentosa (NARP), which often present with severe neurological symptoms owing to the nervous system’s heavy reliance on OXPHOS [71]. These pathogenic mutations frequently arise spontaneously during mtDNA replication or are maternally inherited [72,73,74,75]. Many other mitochondrial syndromes arise from mutations in nuclear-encoded mitochondrial genes, such as POLG [76]. As POLG’s activities span replication, repair, and disease pathogenicity, our review will continually highlight its diverse roles across mitochondrial genome maintenance pathways. Disease-causing mutations in nuclear-encoded genes, such as POLG, are typically transmitted in an autosomal dominant or recessive pattern, resulting in defective mtDNA replication and repair following import of the abnormal polymerase into mitochondria [76,77].

In contrast to these inherited disorders, mtDNA damage most relevant to aging and neurodegeneration, especially those affecting post-mitotic neurons, is accumulated gradually over a lifetime [4,78,79,80]. In this context, mitochondria are consistently exposed to endogenous and environmental sources of reactive oxygen and nitrogen species. The origins of these species, the mtDNA lesions they cause, their respective frequencies, and their connections to neurodegeneration are summarized in Table 1 [18,66,67,81,82]. Chronic exposure to ROS can cause mutagenic lesions and lead to deletions in or loss of mitochondrial genes [69,83]. These cumulative changes can lead to progressive mtDNA instability, which, instead of manifesting as rare inherited syndromes, results in persistent metabolic failure leading to neurodegeneration. Neuronal tissue is therefore acutely sensitive to mitochondrial dysfunction stemming from both inherited and acquired mtDNA instability. Thus, maintaining mtDNA integrity is essential for long-term neuronal survival. Among all genome maintenance mechanisms, BER is the key process safeguarding the mitochondrial genome against the damaging effects of oxidative stress. The degree to which BER preserves mtDNA stability and neuronal viability underlies much of the neurodegenerative risk discussed in subsequent sections.

## 3. Base Excision Repair in Mitochondria: Mechanisms and Enzymes

The persistent oxidative environment, associated with proximity to the ETC, gives rise to a broad spectrum of DNA lesions, including oxidative base modifications, deamination, and alkylation [85]. If left unrepaired, this damage disrupts essential mitochondrial processes and compromises cellular homeostasis [86,87]. To protect against these threats, mitochondria utilize BER, an evolutionarily conserved pathway that identifies chemically altered bases and restores DNA integrity by removing and replacing them [25].

### 3.1. Lesion Detection and Removal

The first and defining step of BER is damage recognition [25,86,88,89]. Because oxidative stress generates a wide variety of chemically modified bases, mitochondria rely on a set of lesion-specific DNA glycosylases to detect these abnormalities with high sensitivity [25,90]. Glycosylases continually scan the genome, probing each base for subtle structural distortions or the loss of chemical functionality [89,90,91]. Once a damaged base is identified, a DNA glycosylase recognizes and excises the lesion, cleaving the N-glycosidic bond to remove the altered base and generate an abasic site; the precise recognition and excision mechanism varies among individual BER glycosylases, which can use distinct lesion-flipping, active-site, and catalytic strategies [89,90,92]. These actions commit the lesion to repair and hand the substrate off to downstream enzymes that complete the restoration of the DNA backbone. Of the eleven mammalian DNA glycosylases, at least seven have confirmed or strongly supported mitochondrial localization and known function within the organelle [25]. These seven mtDNA glycosylases are distributed between all four mammalian glycosylase superfamilies: the uracil-DNA glycosylases (UDG), helix–hairpin–helix (HhH) superfamily, formamidopyrimidine (Fpg)/endonuclease eight (Nei) superfamily, and the alkyladenine DNA glycosylase (AAG) superfamily [25].

The UDG family includes three glycosylases that remove uracil arising from cytosine deamination or misincorporation [93]. The key mitochondrial member of this family is uracil DNA glycosylase isoform 1 (UNG1), which excises uracil and 5-fluorouracil generated by cytosine deamination or deoxyuridine triphosphate (dUTP) misincorporation, with high efficiency on single-stranded DNA and replication intermediates, making it the primary mitochondrial uracil-removal enzyme [93,94].

AAG, also known as methyl purine glycosylase (MPG), is the only member of this mammalian DNA glycosylase superfamily [95]. AAG has been reported to localize to mitochondria via an N-terminal targeting sequence; however, the extent of its mitochondrial contribution may vary among cell types [25,96]. AAG recognizes and removes a broad spectrum of alkylated and deaminated bases, including 3-methyladenine, 7-methylguanine, hypoxanthine, and etheno-adducts, in both single- and double-stranded DNA, providing defense against alkylation and lipid peroxidation-derived lesions within mtDNA [96,97].

The helix–hairpin–helix (HhH) superfamily comprises enzymes that eliminate a wide range of oxidative lesions [98,99]. Among the four members of this superfamily, three localize within the mitochondria: endonuclease three (Nth) like DNA glycosylase 1 (NTHL1), 8-oxoguanine glycosylase (OGG1), and the MutY DNA glycosylase (MUTYH) [25]. NTHL1 and OGG1 remove oxidized pyrimidines and 8-oxoguanine (8-oxoG), respectively [100,101]. NTHL1 removes oxidized pyrimidines, such as thymine glycol, 5-hydroxycytosine, and 5-hydroxyuracil, through its bifunctional glycosylase-lyase activity, thereby counteracting oxidative pyrimidine damage in mtDNA [100]. OGG1 primarily excises 8-oxoG and the related purine lesion FapyG, preventing mutagenic G-to-T transversions that would otherwise accumulate under mitochondrial oxidative stress [101]. MUTYH removes adenine mispaired with 8-oxoG and other oxidized purines, forming an essential component of the OGG1-MUTYH cooperative system that prevents G-to-T mutations in the mitochondrial genome [102].

Lastly, although humans lack a direct ortholog of bacterial Fpg, within the Fpg/Nei superfamily they express three Nei-like (NEIL) enzymes, NEIL1, NEIL2, and NEIL3, which possess distinct subcellular localizations and substrate profiles [103,104,105]. Notably, NEIL1 and NEIL2 function in both mitochondria and the nucleus, whereas studies thus far show that NEIL3 localization and functions are restricted to the nucleus [25]. NEIL1 excises a wide array of replication-blocking oxidative lesions, including thymine glycol (Tg), FapyG, FapyA, and hydantoin derivatives such as spiroiminodihydantoin (Sp) and guanidinohydantoin (Gh), with strong activity on single-stranded and forked DNA structures characteristic of mitochondrial replication intermediates [103,106,107,108,109]. NEIL2 preferentially targets oxidized pyrimidines, such as 5-hydroxyuracil and cytosine-derived lesions, and functions efficiently on bubble and transcription-associated DNA structures relevant to mitochondrial gene expression [104,109,110].

The monofunctional glycosylases that translocate to mitochondria (AAG, MUTYH, and UNG1) possess glycosylase activity only and generate abasic sites [24]. In contrast, the bifunctional glycosylases (NTHL1, OGG1, NEIL1, NEIL2) have intrinsic lyase activity, enabling them to cleave the DNA backbone at abasic sites and generate 3′-blocking ends [24]. After glycosylases remove a damaged base and create an abasic (AP) site or nick the backbone, subsequent end processing is necessary to allow accurate DNA synthesis and ligation.

### 3.2. End Processing

Once a damaged base has been removed, the second phase of BER is carried out by apurinic/apyrimidinic endonuclease 1 (APE1) or polynucleotide kinase/phosphatase (PNKP), which can process the DNA backbone immediately adjacent to the lesion to generate a single-stranded break with ligatable termini [111,112,113]. In monofunctional BER, APE1 incises the abasic site and removes blocking 3′-sugar residues to create a 3′-hydroxyl end suitable for gap filling [98]. In contrast, many bifunctional DNA glycosylases generate strand breaks with 3′-phosphate or 3′-phospho-α,β-unsaturated aldehyde groups that are poor substrates for polymerases and therefore require PNKP to restore a 3′-hydroxyl before ligation [98]. These end processing enzymes ensure that the ends of the DNA are correctly configured for repair synthesis by removing blocking groups, trimming residual sugar fragments, and producing a clean 3′-hydroxyl group required for nucleotide insertion by a DNA polymerase. During long-patch (LP) BER in the mitochondria, 5′-exo/endonuclease G (EXOG) is the primary enzyme that further processes the displaced flap to create a nick suitable for ligation [114,115,116]. When EXOG activity is limited, or when the lesion is inhibitory to EXOG, 5′-flap endonuclease 1 (FEN1) can provide supplementary 5′-end processing, thereby offering a secondary LP-BER route for excising flaps or damaged 5′ termini that are suboptimal substrates for EXOG [28,32,114,117]. By converting the AP site into a repair-ready substrate, this step maintains the pathway’s continuity and prevents accumulation of unstable intermediates that would otherwise threaten genome stability.

### 3.3. Gap Filling

With properly processed DNA ends, the next stage of BER restores the missing nucleotides. In both the nucleus and mitochondria, gap filling occurs via two subpathways of BER: short-patch (SP) and LP-BER, which differ in the number of nucleotides inserted, the enzymes involved, and the types of lesions they resolve. SP-BER incorporates a single nucleotide and is generally sufficient for simple, non-bulky lesions. In contrast, LP-BER inserts 2–7 nucleotides, and together with a 5′-end processing-nuclease, removes an oxidized or otherwise unligatable 5′ terminus, enabling repair of more complex lesions such as oxidized AP sites [25,26,117,118]. In mitochondria, gap filling is primarily carried out by polymerase gamma (POLG), which supports BER by inserting nucleotide(s) into the repair gap before ligation [119,120]. A fraction of polymerase beta (POLB), the main gap-filling enzyme in nuclear BER, also localizes to the mitochondria to provide additional gap-filling and deoxyribose phosphate (dRP)-lyase capacity. POLB is particularly important for processing BER intermediates that contain 5′-dRP groups or when POLG becomes limiting [121,122,123].

### 3.4. Ligation

The final phase of BER restores DNA strand continuity by ligating the broken strand. Once the correct nucleotide(s) have been inserted by SP or LP processes, DNA ligase IIIα (LIG3), the principal DNA ligase operating in mitochondria, seals the remaining nick in the phosphodiester backbone, completing the repair process [124]. LIG3 ligates not only BER-generated nicks in mtDNA but also serves an important role during mtDNA replication, helping to close newly synthesized genomes into intact circles, thereby reinforcing its predominant role in mtDNA ligation [125]. By closing the repair intermediate and fully reestablishing strand integrity, ligation prevents single-strand breaks from persisting, thereby avoiding replication stalling, double-strand break formation, and mitochondrial genome instability, and allowing the mtDNA molecule to resume normal replication and transcription.

## 4. BER Dysfunction: A Delicate Balance in Mitochondrial Genome Maintenance

**Figure 2:**
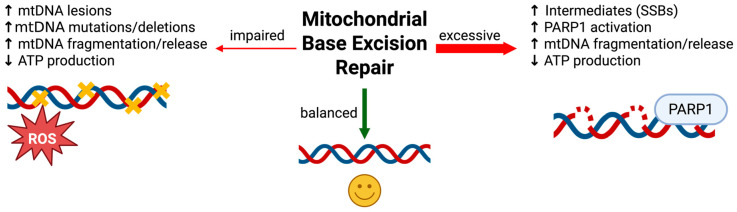
Schematic of mitochondrial base excision repair (BER) activity levels and their respective cellular outcomes. Balanced BER maintains mitochodnrial DNA (mtDNA) integrity. Impaired BER leads to accumulation of mtDNA lesions, genome instability, and energy failure. Excessive BER generates toxic repair intermediates, triggers poly(ADP-ribose) polymerase 1 (PARP1) overactivation, genome instability, and energy failure.

Understanding the crucial role of mitochondrial BER in maintaining neuronal genome stability and energy balance helps set the stage for evaluating the serious consequences that occur when this pathway fails. Impaired mitochondrial BER arises through multiple, often convergent mechanisms: age-related declines in BER enzyme expression, chronic oxidative stress that exceeds repair capacity, and mutations or epigenetic modifications in nuclear genes encoding BER proteins or proteins required for their mitochondrial import. Paradoxically, both insufficient and excessive BER activity can be detrimental to cellular health (Figure 2).

Loss or reduced activity of mitochondrial BER enzymes directly leads to mtDNA instability, as demonstrated across a broad range of animal models and in vitro studies. In mice, knockout of NEIL1 results in accumulation of mtDNA mutations and deletions, leading to a metabolic syndrome phenotype and increased oxidative sensitivity [126]. OGG1-deficient mice display elevated levels of 8-oxoG throughout mitochondrial genomes in multiple tissues compared to wild-type controls [127]. Silencing or mutating mitochondrial BER proteins, including OGG1, NEIL1, MUTYH, and POLG, in diverse cell models, including but not limited to neuronal systems, results in substantial accumulation of oxidized base lesions, increased mtDNA mutations and deletions, increased genome instability, and diminished mitochondrial respiration in response to oxidative challenges [128,129,130,131].

In neuronal systems specifically, neural stem cells (NSCs) isolated from OGG1-deficient mice show increased mtDNA 8-oxoG accumulation, impaired neuronal differentiation, and reduced neurite outgrowth after oxidative challenge compared to NSCs from wild-type mice [132]. These and other BER-compromised neural models exhibit a characteristic constellation of mitochondrial defects, including diminished basal and maximal respiration, decreased ATP production, mitochondrial membrane depolarization, and heightened susceptibility to apoptosis following ROS exposure [131,132]. In primary neurons and brain tissue from BER-deficient mice, these mitochondrial deficits are accompanied by synaptic loss, altered firing properties, and region-specific neurodegeneration, indicating that even moderate, chronic BER impairment can deplete the bioenergetic reserves of vulnerable neuronal populations and trigger progressive cell loss [131,132]. Together, these findings demonstrate that failure of mitochondrial BER drives persistent mtDNA instability and bioenergetic collapse in neuronal contexts, creating a feed-forward loop in which defective repair promotes mitochondrial dysfunction, mtDNA release, and neuroinflammatory signaling that converge on neurodegenerative disease. The consequences of impaired BER are especially evident when defects extend to widespread loss of some BER proteins. Biallelic loss of NTHL1 or MUTYH results in NTHL1-associated polyposis (NAP) and MUTYH-associated polyposis (MAP), respectively [133,134,135]. These are hereditary cancer disorders characterized by high colorectal adenoma burden and increased cancer risk, particularly colorectal cancer [133,134,135]. The severity of these syndromes underscores the essential role of BER in safeguarding genome integrity and preventing malignant transformation.

However, excessive or unregulated BER can also be deleterious. The natural progression of the BER pathway generates strand-break intermediates when lesion recognition and base excision occur faster than gap filling and ligation, producing toxic repair intermediates that accumulate and become a source of genome instability rather than protection [136]. Within mitochondria, where repair enzyme stoichiometry and nucleotide availability are tightly constrained, this imbalance can stall repair, collapse replication forks, and fragment the mitochondrial genome. In this context, mitochondrial poly(ADP-ribose) polymerase 1 (PARP1) has been proposed to be recruited to BER intermediates and, when overactivated or present in excess, can clamp onto mtDNA breaks, consume NAD^+^, and inhibit POLG-dependent gap filling, thereby exacerbating mtDNA damage and impairing mitochondrial function [137]. While the localization and function of PARP1 within mitochondria remain debated, primary studies provide key insights into these discrepancies. Proximity ligation assays (PLA), an in situ method, detected PARP1 interactions with mitochondrial BER enzymes EXOG and POLG in A549 cells [138]. PARP1 and EXOG/POLG interactions were observed under resting conditions and increased in response to oxidative stress [138]. In contrast, confocal microscopy of eGFP-tagged PARP1in HeLaS3 cells and mouse fibroblasts showed no mitochondrial colocalization despite strong nuclear signal, raising questions about whether tagging disrupts potential mitochondrial import and cell type differences [139]. Overexpression of several BER enzymes, including the DNA glycosylases AAG, OGG1, and NEIL1, has been shown to increase DNA damage intermediates [140,141,142]. At the same time, targeted overexpression of mitochondrial OGG1 can enhance mtDNA repair capacity, limit oxidant-induced mtDNA damage and apoptosis, and improve mitochondrial function and metabolic homeostasis in specific settings, illustrating that increased glycosylase activity can be beneficial when matched to the relevant lesion burden [143]. These repair intermediates can trigger innate immune sensors and further amplify inflammatory signaling [142,144,145,146,147,148].

Taken together, proper coordination and proportional activity within the BER pathway are critical for mtDNA homeostasis. Insufficient BER allows oxidative lesions to persist, promoting mtDNA instability and chronic inflammation, whereas excessive BER generates an excess of repair intermediates that are equally destabilizing and immunostimulatory. Neurons are particularly vulnerable to mtDNA instability because of their high metabolic rate. As previously discussed, mature neurons are post-mitotic and cannot re-enter the cell cycle or be easily replenished in response to injury or during an energy crisis. Their survival and function depend on the careful regulation of mitochondrial homeostasis, including spatial distribution at synaptic sites and the continuous production of ATP required for neurotransmission, maintenance of ion gradients, and intracellular transport [1,2]. Due to these metabolic demands, neurons are especially sensitive to disruptions in mitochondrial OXPHOS. Loss or mutation of mtDNA-encoded subunits can impair ETC assembly and function, leading to decreased ATP production, increased oxidative stress, and the activation of degenerative pathways [85]. Because neurons cannot readily compensate for mitochondrial dysfunction, chronic bioenergetic deficits lead to synaptic dysfunction, reduced plasticity, and eventual cell death, making mtDNA instability a critical driver of neurodegenerative disease in the central nervous system.

## 5. mtDNA Instability as a Trigger for Neuroinflammation

**Figure 3:**
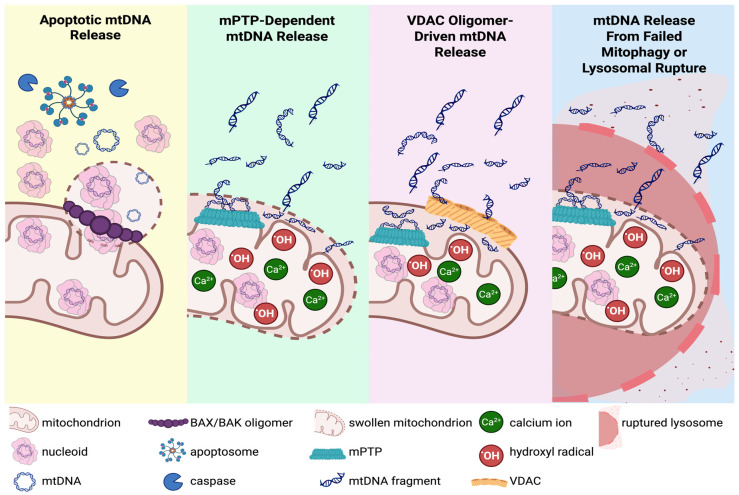
Mechanisms of mitochondrial DNA (mtDNA) release under cellular stress conditions. Apoptotic mtDNA release: Mitochondrial outer membrane permeabilization (MOMP) is induced by the pro-apoptotic BCL-2 family proteins BAX/BAK macropore formation during apoptosis, enabling the escape of mtDNA and entire nucleoids into the cytosol; mPTP-dependent mtDNA release: The mitochondrial permeability transition pore (mPTP) opens in response to excessive calcium or oxidative stress, allowing small mtDNA fragments to exit the matrix. Persistent mPTP opening leads to mitochondrial swelling and outer membrane rupture, facilitating the release of matrix contents into the cytosol; VDAC oligomer-driven mtDNA release: Under moderate cellular stress, voltage-dependent anion channel (VDAC) oligomerization promotes the release of small mtDNA fragments into the cytosol. This process requires prior permeabilization of the inner membrane, often via mPTP; mtDNA release from failed mitophagy or lysosomal rupture: Ineffective removal of damaged mitochondria by mitophagy or lysosomal rupture results in the leakage of mtDNA from permeabilized or ruptured organelles into the cytosol.

In addition to impairing neuronal bioenergetic capacity, growing evidence shows that mitochondrial genome instability can lead to the release of mtDNA fragments, which act as potent DAMPs that trigger and amplify neuroinflammatory signaling [149,150,151,152,153,154]. Several stress-response pathways enable mtDNA to escape the mitochondrial matrix and reach the cytosol or the extracellular space of the neuron, linking impaired mitochondrial quality control directly to inflammatory responses within the nervous system (Figure 3).

During severe mitochondrial stress or injury, the mitochondrial outer membrane becomes permeable (MOMP), often mediated by the pro-apoptotic BCL-2 family proteins BAX and BAK, which oligomerize into macropores during apoptosis and enable the extrusion of entire mitochondrial nucleoids or DNA fragments into the cytosol [155,156]. This breach has been observed during caspase-dependent and caspase-independent apoptosis and in certain non-apoptotic stress responses, indicating that mtDNA release via MOMP is not restricted to terminal cell death [156]. Excessive calcium or oxidative stress can induce opening of the mitochondrial permeability transition pore (mPTP), causing matrix swelling and inner-membrane permeabilization [157]. This permeabilization provides a route for smaller mtDNA fragments to cross the inner membrane into the intermembrane space and, ultimately, the cytosol [149]. Depending on the duration and extent of opening, mPTP activation can function as a reversible stress response or progress to catastrophic mitochondrial dysfunction and cell death [157]. Under more moderate stress, oligomerization of the voltage-dependent anion channel (VDAC) in the outer membrane has been proposed to support mtDNA fragment release without complete mitochondrial rupture or overt apoptosis [158]. In addition to membrane permeabilization, defects in mitochondrial turnover, including impaired mitophagy or lysosomal degradation, can allow damaged mitochondria to persist and eventually rupture, leading to mtDNA leakage even in the absence of apoptosis [159,160,161,162,163]. Thus, mitochondrial injury, disrupted clearance, or incomplete organelle degradation each create opportunities for mtDNA to escape regular compartmentalization. The specific location of mtDNA release (cytosol or extracellular matrix) determines which immune pathways are activated and how neuroinflammatory signaling is propagated throughout the nervous system.

Once in the neuron’s cytosol, mtDNA fragments activate robust intrinsic immune pathways and inflammasome signaling, which are closely coupled to mitochondrial dysfunction and neuronal death. The cGAS-STING pathway is a key mechanism for detecting cytosolic mtDNA. cGAS binds cytosolic DNA to produce cGAMP, which activates STING and triggers transcriptional responses to stimulate interferon and proinflammatory cytokine production [34,154,164]. Emerging evidence indicates that oxidized forms of mtDNA are particularly immunostimulatory and more efficiently activate STING signaling [165]. Additionally, oxidized or fragmented mtDNA can directly engage inflammasome complexes, particularly NLR (NOD-like receptor) Family Pyrin Domain Containing 3 (NLRP3), leading to caspase-1 activation and the production of Interleukin-1 beta (IL-1β) and other cytokines, further amplifying inflammation and contributing to local tissue damage [166].

**Figure 4:**
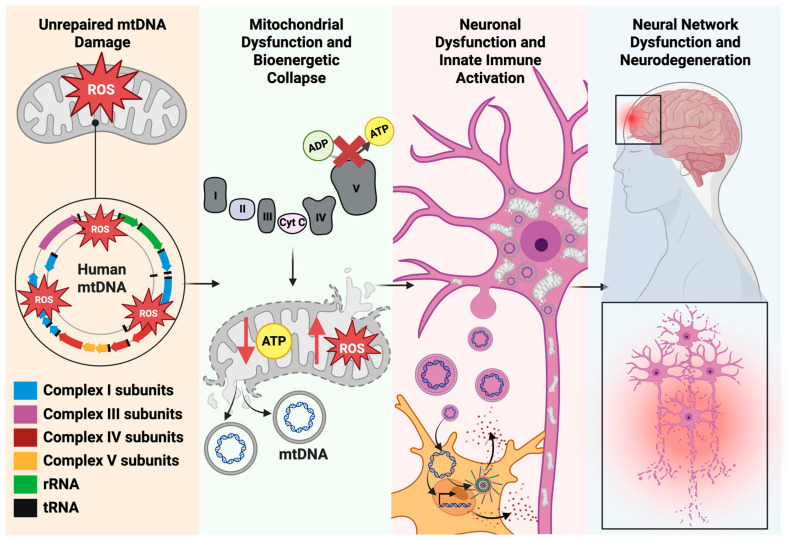
Cascade of Neurodegeneration Initiated by mitochondrial DNA (mtDNA) Damage. Unrepaired mtDNA damage in neurons impairs electron transport chain function, reduces ATP production, and increases reactive oxygen species (ROS). Damaged mitochondria accumulate when mitophagy is impaired or overwhelmed, further elevating ROS levels and releasing mtDNA fragments. These fragments act as damage-associated molecular patterns (DAMPs) that activate microglial innate immune pathways, driving neuroinflammation, neuronal dysfunction, and progressive neurodegeneration.

mtDNA DAMP signaling extends beyond the cytosol of the originating neuron. Damaged neurons can actively expel mtDNA into the extracellular space via vesicle secretion, mitochondrial extrusion, or direct membrane rupture [152,167]. Cell-free mtDNA species can then be absorbed by neighboring glial cells, the major initiators of widespread neuroinflammation. As the brain’s resident immune cells, microglia are especially sensitive to extracellular mtDNA. Microglia rapidly phagocytose extracellular mtDNA, triggering a potent cGAS-STING pathway and inflammasome activation, amplifying neuroinflammation far beyond neuronal responses by secreting high levels of tumor necrosis factor-alpha (TNFα), interleukin-6 (IL-6), and interferon signaling [168,169]. Astrocytes respond to mtDNA primarily through toll-like receptor 9 (TLR9) [170]. TLR9 binds the mtDNA which activates nuclear factor kappa-light-chain-enhancer of activated B cells (NF-κB) to initiate transcription of IL-6, IL-1β, C-C motif chemokine ligand (CCL2), and C-X-C motif chemokine ligand 10 (CXCL10) [170]. Ultimately, persistent or recurrent mtDNA DAMP signaling establishes a self-perpetuating cycle: mitochondrial dysfunction promotes neuroinflammation, while inflammatory mediators further damage mitochondria and destabilize mtDNA. This reciprocal cycle establishes a direct molecular connection between impaired mtDNA repair, chronic innate immune activation, disrupted neuronal health, and accelerated neurodegenerative decline [168,169,171] (Figure 4).

Together, these mechanisms highlight a direct molecular link between defective mtDNA maintenance, from oxidative damage, impaired repair, or failed organelle quality control, and pathological inflammation. This connection is increasingly recognized as a key driver in the progression of disorders such as Alzheimer’s disease, Parkinson’s disease, traumatic brain injury, and other neuroinflammatory and neurodegenerative conditions.

## 6. Emerging Therapeutic Strategies

Therapeutic strategies aimed at limiting mtDNA instability can intervene at multiple levels: by directly supporting mitochondrial BER, by improving the mitochondrial environment in which BER operates, and by attenuating the downstream inflammatory and metabolic consequences of BER failure. In rodent models, delivery of BER enzymes, such as OGG1 or APE1, to neurons using adeno-associated virus (AAV)-mediated gene transfer and transgenic or mitochondrially targeted overexpression has been shown to reduce mtDNA damage, improve mitochondrial function, and restore neural viability [172,173]. Strategies developed for primary mitochondrial diseases can inform treatment approaches for neurodegenerative and neuroinflammatory conditions, in which mtDNA instability frequently arises secondary to aging-related oxidative stress and inflammation [174]. For example, nucleoside supplementation strategies developed for primary mtDNA maintenance disorders are discussed as a template for treating more common conditions, including neurodegenerative states [174,175,176]. Supplementation with nucleotides or their precursors may counteract nucleotide pool imbalances and support efficient repair and replication, particularly in conditions where POLG function is compromised [175,176].

The development of genome-editing tools further enables correcting pathogenic mtDNA mutations or removing mutant mtDNA genomes altogether, thereby reducing mitochondrial heteroplasmy and restoring the pool of healthy mtDNA [177,178,179,180,181,182,183,184]. Experimental mitochondrial transplantation and intercellular mitochondria transfer approaches likewise seek to repopulate stressed or dysfunctional neurons with respiration-competent organelles, improving bioenergetics and attenuating tissue injury in preclinical models [185,186,187,188,189,190,191,192,193]. Despite their promise, mitochondrial genome editing approaches raise important safety concerns, including potential off-target cleavage or deamination events, editing of low-level wild-type genomes, and unanticipated effects on nuclear DNA [181]. Similarly, mitochondrial transplantation strategies face challenges related to immunogenicity and controlled integration into host networks [185]. Further, mitochondria-tagged antioxidants (MTAs) reduce the oxidative burden on mtDNA and mitigate the formation of lesions that overwhelm BER capacity [194].

Therapeutic innovation is also targeting the downstream consequences of BER failure. Restoring global mitochondrial quality control is a significant focus, with compounds that stimulate mitophagy or promote mitochondrial biogenesis showing promise in reducing the accumulation of mutant mtDNA and limiting the release of proinflammatory signals [195,196,197,198,199,200,201,202]. However, while stimulated mitophagy generally protects against the accumulation of damaged mitochondria and proinflammatory signaling, excessive mitophagy has also been linked to programmed neuronal death and increased neuroinflammation in preclinical models, highlighting the need for careful modulation and therapeutic balance [203]. Inhibitors of the cGAS-STING pathway and the NLRP3 inflammasome are now being assessed in preclinical models for their ability to blunt DAMP-induced neuroinflammation [204,205,206,207,208,209,210,211,212]. PARP1 inhibitors provide an additional means to mitigate metabolic collapse during unbalanced BER by limiting PARP1-dependent consumption of NAD^+^ and ATP, which can secondarily compromise mitochondrial bioenergetics, and by potentially modulating PARP1 activity reported at mtDNA lesions [137,138,213,214,215,216,217].

Although many of these interventions remain at preclinical or early translational stages, they collectively highlight the multifaceted therapeutic opportunities that arise from understanding how mtDNA instability drives neurodegeneration. Ultimately, a combinational approach that directly augments mitochondrial BER, protects from oxidative challenge, and modulates maladaptive neuroimmune responses is likely to be most effective for restoring mitochondrial homeostasis and preventing neuronal loss in neurodegenerative conditions.

## 7. Conclusions and Future Directions

Mitochondrial BER stands as a fundamental guardian of neuronal genome stability and energy homeostasis, particularly in the context of the central nervous system’s extraordinary metabolic demands and long-lived post-mitotic cells [24,25,26,27,79,86]. Decades of research have clarified that both insufficient and excessive mitochondrial BER disrupt the delicate balance required for effective DNA repair, leading to persistent mtDNA instability, bioenergetic collapse, and aberrant activation of innate immune pathways [126,127,128,129,130,131,132]. These consequences, amplified by the neural tissue’s unique vulnerabilities, form a mechanistic bridge connecting inherited and acquired mtDNA defects to the pathogenesis of neurodegenerative and neuroinflammatory diseases.

Recent advances in molecular genetics, neuroimmunology, and mitochondrial medicine have expanded the therapeutic landscape, offering new ways to intervene at multiple points along the mtDNA damage-response spectrum. Gene and enzyme delivery approaches, mitochondrial genome editing, targeted antioxidants, mitochondrial transplantation, and precision immunomodulators provide promising routes to restore mitochondrial stability or limit downstream pathology [138,172,173,174,175,176,177,178,179,180,181,182,183,184,185,186,187,188,189,190,191,192,193,194,195,196,197,198,199,200,201,202,203,204,205,206,207,208,209,210,211,212,213,214,215,216,217]. However, meaningful clinical translation will require surmounting several challenges. Achieving neuronal and region-specific delivery, tuning repair activity without triggering harmful hyperactivation, and establishing biomarkers that reliably monitor mtDNA repair capacity and innate immune engagement in vivo will be essential steps. Likewise, integrating heterogeneity in neuronal subtypes, aging trajectories, and metabolic states into therapeutic design will be crucial for identifying which interventions are most beneficial and then deploying them.

Several outstanding research questions define the path ahead. How can BER activity be precisely calibrated to maintain fidelity without tipping into detrimental overactivity? Are there biomarkers for early mtDNA instability, and can they predict therapeutic windows before irreversible neurodegeneration begins? A recently developed blood-based mtDNA damage detection assay (Sanders and colleagues) stratifies Parkinson’s disease patients, and illustrates that such mtDNA-focused biomarkers are feasible and could be adapted to other neurodegenerative settings [84]. Further, which immune signaling pathways downstream of mtDNA DAMPs are most amenable to therapeutic modulation without unintended immunosuppression? And how do individual genetics, metabolic states, and mitochondrial dynamics shape the susceptibility of neurons to BER imbalance, mitochondrial quality control, and neuroimmune activation? Addressing these gaps will depend on multidisciplinary collaboration, the development of innovative tools, and seamless translation from experimental models to human systems. Ultimately, restoring the balance of mitochondrial BER and limiting pathological mtDNA instability holds the potential to significantly slow, or perhaps prevent, the progression of multiple neurodegenerative and neuroinflammatory disorders. By uniting insights from DNA repair, mitochondrial biology, and neuroimmunology, the field is poised to develop interventions that address the root cause of neuronal decline rather than its downstream consequences.

## 8. Literature Search and Selection

Literature for this narrative review was identified primarily through PubMed and Google searches using combinations of keywords related to mitochondrial DNA damage, base excision repair, mitochondrial dysfunction, neuroinflammation, and neurodegeneration. We focused on peer-reviewed original research and review articles and included both seminal foundational studies and recent work. One preprint is cited and clearly indicated in the text where it provides timely mechanistic insights that extend published findings. AI tools such as ChatGPT 5.2 and Perplexity were used to help with identifying grammatical errors and issues with sentence structure.

## Figures and Tables

**Table 1:** Major mitochondrial reactive oxygen/nitrogen species sources and mtDNA lesions relevant to neurodegeneration.

Source	Primary Species	mtDNA Lesion Types/Frequency *	Neurodegeneration/Neuroinflammation Links
ETC complexes I/III [67]	Superoxide, hydrogen peroxide [67]	Oxidized base lesions (e.g., 8-oxo-dG, FapyG, thymine glycol), abasic sites; contributes to steady-state mtDNA damage under basal conditions, with elevated lesion burden in aging and neurodegenerative models [66]	Contributes to mitochondrial dysfunction and mtDNA damage in Parkinson’s, Alzheimer’s, and other neurodegenerative disorders [67]
Mitochondrial dehydrogenases (e.g., α-ketoglutarate dehydrogenase) [67]	Superoxide, hydrogen peroxide [67]	Oxidized base lesions, abasic sites; contributes to steady-state mtDNA damage under basal conditions, with elevated lesion burden in aging and neurodegenerative models [66]	Altered dehydrogenase activity and elevated ROS are reported in aging brain and in models of neurodegenerative disease [67]
Neuroinflammation (microglial/astrocytic inducible nitric oxide synthase) [81]	Nitric oxide, peroxynitrite [81]	Increased oxidized base lesions, abasic sites, strand breaks; mtDNA lesion burden often reported as significantly higher than basal levels in inflammatory states [81]	Chronic neuroinflammation contributes to mitochondrial dysfunction and neuronal loss [81]
Transition metal dyshomeostasis (e.g., iron) [82]	Hydroxyl radical via Fenton chemistry [82]	Increased oxidized base lesions, abasic sites, strand breaks; lesion burden increased relative to basal conditions, particularly in metal-rich brain regions [82]	Metal accumulation and mitochondrial oxidative damage are implicated in Parkinson’s disease, Alzheimer’s disease, and other neurodegenerative disorders [82]
Xenobiotic mitochondrial toxins (e.g., rotenone) [84]	Superoxide, hydrogen peroxide [84]	Increased oxidized base lesions, abasic sites, strand breaks, large-scale deletions; experimental models frequently report marked increases in mtDNA damage [84]	ETC toxins are classic Parkinson’s models, where mitochondrial ROS, mtDNA damage, and dopaminergic neuron loss are tightly coupled [84]

* Note: Many mtDNA lesions (8-oxo-dG, FapyG, thymine glycol, abasic sites, strand breaks) are common end products of multiple reactive oxygen/nitrogen species sources. As a result, lesion spectra often overlap substantially, and reported basal levels (typically a few lesions per 10^6^ nt of mtDNA in mammalian brain tissue) reflect cumulative oxidative and nitrative damage rather than source-specific signatures.

## Data Availability

No new data were created or analyzed in this study.
